# Send more data: a systematic review of mathematical models of antimicrobial resistance

**DOI:** 10.1186/s13756-018-0406-1

**Published:** 2018-09-29

**Authors:** Anna Camilla Birkegård, Tariq Halasa, Nils Toft, Anders Folkesson, Kaare Græsbøll

**Affiliations:** 1 Department of Applied Mathematics and Computer Science, Technical University of Denmark, Asmussens Allé Building 303B, 2800 Kgs. Lyngby, Denmark; 2 Division of Diagnostics & Scientific Advice, Technical University of Denmark, Kemitorvet Building 204, 2800 Kgs. Lyngby, Denmark; 3 Department of Biotechnology and Biomedicine, Technical University of Denmark, Kemitorvet Building 204, 2800 Kgs. Lyngby, Denmark

## Abstract

**Background:**

Antimicrobial resistance is a global health problem that demands all possible means to control it. Mathematical modelling is a valuable tool for understanding the mechanisms of AMR development and spread, and can help us to investigate and propose novel control strategies. However, it is of vital importance that mathematical models have a broad utility, which can be assured if good modelling practice is followed.

**Objective:**

The objective of this study was to provide a comprehensive systematic review of published models of AMR development and spread. Furthermore, the study aimed to identify gaps in the knowledge required to develop useful models.

**Methods:**

The review comprised a comprehensive literature search with 38 selected studies. Information was extracted from the selected papers using an adaptation of previously published frameworks, and was evaluated using the TRACE good modelling practice guidelines.

**Results:**

None of the selected papers fulfilled the TRACE guidelines. We recommend that future mathematical models should: a) model the biological processes mechanistically, b) incorporate uncertainty and variability in the system using stochastic modelling, c) include a sensitivity analysis and model external and internal validation.

**Conclusion:**

Many mathematical models of AMR development and spread exist. There is still a lack of knowledge about antimicrobial resistance, which restricts the development of useful mathematical models.

## Background

The discovery of antimicrobials in medicine in the 1920s was regarded as a miracle. Since then, millions of lives have been saved as a result of this treatment. However, history has shown that the introduction of any kind of antimicrobial compound into human or veterinary medicine is swiftly followed by emerging resistance to that compound [1]. Antimicrobial resistance (AMR) is threatening our ability to treat common infectious diseases, resulting in prolonged illness, disability and death [2]. Multidrug and even pan-resistant organisms are now a worldwide problem. Despite the difficulty in estimating the actual costs of AMR, the true economic burden is substantial [3]. The estimated economic consequences of AMR in Europe in 2007 were at least €1.5 billion, while they were estimated to be $55 billion in the US in 2000 (cited from Gandra et al., 2014 [3]). It is therefore of utmost importance to limit the emergence and spread of AMR.

AMR is spreading globally - not just in the human population, but also in animal populations and the environment. Furthermore, there is consistent evidence that an exchange of bacteria resistant to antimicrobials and AMR determinants exists between these different compartments [4]. AMR determinants have been shown to survive in environments such as sludge and wastewater treatment systems [5, 6], thus allowing for the transmission of infectious bacteria and accelerating the problem of AMR.

Mathematical models have become important decision support tools in medicine and public health [7]. They have helped in improving our understanding of the development, emergence and spread of AMR [7, 8]. In addition, they can identify gaps in our knowledge, and direct research towards missing information for important parameters and processes in the modelled system. However, in 2006, Opatowski et al. [7] published a review on mathematical models on AMR and concluded that there was still a need for major improvements of AMR models such as regarding implementing important features of pathogen including resistance mechanisms and inter-species cooperation. Continual evaluation of published mathematical models is therefore necessary for us to recognise the progress in AMR modelling. Gaps in our knowledge can be identified, and this can be used to set the agenda and form suitable hypotheses for future research in the fight against AMR.

Grimm et al. [9] updated the TRACE paradigm that was established in 2010 with the aim of developing guidelines to produce useful models. The TRACE paradigm includes eight elements that, when followed, ensure that models are clearly communicated when published. These elements are: 1) Problem formulation (clear formulation of the objective and a description of the context of the model); 2) Model description (written description of model elements to allow readers to understand and replicate the model); 3) Data evaluation (an assessment of the quality of data used to parameterise the model); 4) Conceptual model evaluation (a list and explanation of the most important conceptual design decisions); 5) Implementation verification (internal validation of the model, testing for programming errors and assessing model performance); 6) Model output verification (external validation, testing whether the model output matches the observations); 7) Model analysis (mainly sensitivity analysis); 8) Model output corroboration (a comparison of model output with data that were not used to create the model). For a full description of the TRACE elements, see Grimm et al. [9].

Since the comprehensive systematic review of mathematical models between 1993 and 2006 was conducted by Temime et al. [10], a number of additional reviews have been published [7, 8, 11]. However, these reviews either focused on models linking antibiotic use to AMR [11] or modelling AMR in populations (humans and bacteria) and hospitals [7], and did not include exclusively within-host models [8]. These systematic reviews did not examine models of AMR in relation to animal populations and the environment. However, a comprehensive review of mathematical models of AMR should consider models of all relevant populations and ecosystems in order to target the AMR problem from a One-Health perspective. In this way, researchers from different fields could benefit from experiences and advances in the other fields.

The objective of this review was to assess the usefulness of mathematical and simulation models of AMR development and/or spread in individuals and/or populations of humans, animals and bacteria, as well as in the environment. We also aimed to identify gaps in the knowledge needed to provide useful models of AMR. The assessment was achieved using a systematic review. The published models were then summarised and compared using an adapted version of previously developed frameworks [7, 8]. Furthermore, the strengths and weaknesses of the models were discussed using the TRACE paradigm [9] .

## Methods

This is a systematic review following the PRISMA guidelines [12] without the use of an existing review protocol.

### Selection of papers and search criteria

The search was performed in PubMed and Web of Science on 6th February 2017. We used the following search terms:

In PubMed three searches were performed:

1. ((((((((antimicrobial) OR antibiotic) OR antibacterial) AND “last 10 years”[PDat])) AND resistan*) AND model[Title]) AND “last 10 years”[PDat] AND English[lang]).
2. ((((((((antimicrobial) OR antibiotic) OR antibacterial) AND “last 10 years”[PDat])) AND resistan*) AND model[Title]) AND “last 10 years”[PDat] AND English[lang]).

In Web of Science three searches were performed:

1. TS = (resistan*) AND (TS = (antibacterial) OR TS = (antimicrobial) OR TS = (antibiotic)) AND TITLE: (model*) ENGLISH, ARTICLE, REVEIW, 2006–2017 & TS = (resistan*)
2. (TS = (antibacterial) OR TS = (antimicrobial) OR TS = (antibiotic)) AND TITLE: (population dynamic*) ENGLISH, ARTICLE, REVIEW, 2006–2017
3. TS = (resistan*) AND (TS = (antibacterial) OR TS = (antimicrobial) OR TS = (antibiotic)) AND TITLE: (simulat*) ENGLISH, ARTICLE, REVIEW, 2006–2017

**Fig. 1:**
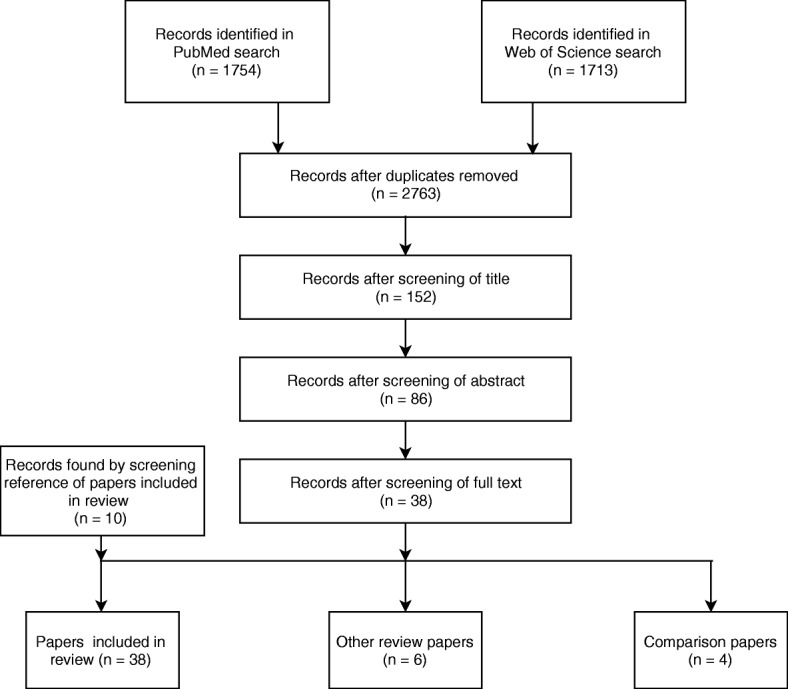
Exclusion tree in the selection of papers

We checked for duplicates between the two databases, and excluded papers based on titles. More papers were excluded after the abstracts of all papers were screened. Finally, following full screening of the papers, some were deemed not to fulfil the inclusion criteria and were therefore excluded (Fig. 1). The screening of abstracts and full papers was carried out by three of the authors, with each person reading 2/3 of the abstracts and 2/3 of the full papers.

Papers were included if they: 1) presented not previous published mathematical models that represented the development and/or spread of AMR; 2) modelled AMR in bacteria, humans, animal or the environment, and 3) included the effect of antimicrobial compounds. Papers that focused on only the spread of a specific resistant bacterial pathogen were not included. In addition, papers based solely on statistical analysis were also excluded, as this review focuses on mathematical models.

Previously published reviews of mathematical models of AMR were identified during the review process. The reference lists of these reviews were scrutinised to identify additional papers that might fit the inclusion criteria. These are referred to as “papers identified by other means”.

### Analysing the papers

Three of the authors read the selected papers. Each person read a random sample of 2/3 of the papers (as described above), while ensuring that each paper was read by two authors. Information about the papers was extracted and sorted into five constructs (model description, modelling technique, modelling pathways, model specifications and model validation).

Relevant information for each of the five constructs was extracted from the papers. This information is described in Table 1. Furthermore, information on the following descriptive parameters was extracted from the papers: programming software, year of the publication of the paper, and the country of affiliation of the first author.

**Table 1:** Description of the information extracted from the selected studies

Construct	Attributes	Levels	Comments
Model description	Modelled process	Spread of AMR Development of AMR Spread and development of AMR	Other types of AMR processes were for example fitness cost and difference in resistance due to the age of bacteria.
	Model type	Agent based Nested model Individual based Population Other models	Other model types were: Beverton-Holt, cellular automata, band-pass, or chemostat model.
	Population	Animal species Bacteria Humans	No specified host was used in cases where only the bacterial population was modelled.
	Environment	River Slurry Cellular automata Community Farm Hospital Human Animal species In vitro Not specified	Hospital refers to both human and veterinary hospitals.
Modelling techniques	Simulation / analytic	Simulation Analytic Analytic & simulation	
	Uncertainty display	Deterministic Stochastic Deterministic & stochastic	Both deterministic and stochastic were used for example in the case of nested models and papers comparing deterministic and stochastic models.
Modelling population interactions	Mixing of population	Homogenous mixing Heterogeneous mixing Homogenous & heterogeneous mixing Not relevant	Both heterogeneous and homogenous mixing refers to e.g. rivers and network models with homogenous mixing at the nodes but heterogeneous between nodes. Not relevant may refer to development of specific traits such as efflux pumps.
	Co-existence level^a^	No conversion Single strain Uni-directional Bi-directional	Uni-directional was defined as a one-way conversion from resistant strain/carrier to sensitive strain/carrier or vice versa, whereas bi-directional conversion was possible in case of a two-way conversion. In case of no conversion, only competition between strains was possible.
Model specification	AMR display	Genotypic AMR Phenotypic AMR Genotypic & phenotypic AMR Other types of AMR	Other types of AMR describing a more molecular AMR mechanism were for example modelling of efflux pumps or plasmids.
	Number of resistant strains	Single resistant strain Multiple resistant strains	Multiple resistance means that two or more strains of the same bacterial species resistant to antimicrobials were modelled
	Bacterial species	Specified Not specified	If specified, the specific species was noted
	Dosing of the antimicrobial	Constant dosing Other dosing	Other dosing was for example specific treatment strategy or spatial distribution
	Antimicrobial compound	Single, not specified Single, specified Multiple, not specified Multiple, specified	If specified, the specific compound was noted
	Immune system	Yes No Not relevant	Not relevant describes situations where the model did not incorporate a human or animal host with a functioning immune system.
Model validation	Model validation	Literature No validation	
	Sensitivity analysis	Yes No	
	Bifurcation analysis	Yes No	

*AMR* Antimicrobial resistance; ^a^the co-existence level was described according to Spicknall et al. [8], modified to population leve

### Model usefulness and documentation

We used the TRACE framework for good practice in model development and documentation developed by Grimm et al. [9] to evaluate the conformity of the models to the TRACE guidelines. These guidelines ensure that useful models are produced.

Studies were initially evaluated according to the constructs described in Table 1. Hereafter, studies that had verified the model and conducted sensitivity analysis – hence complying with two of the TRACE criteria [9] (Criteria 6 and 7) – were identified. These studies were further evaluated to assess whether they fulfilled the remaining criteria.

## Results

### Exclusion of papers

Studies that were excluded based on their title mainly included models of pathogen spread and the mode of action of antimicrobial compounds. The main reason for excluding studies based on the abstract or the full paper was that the study described statistical models of AMR spread and development. Another reason for exclusion was that the studies described a model of resistant bacteria without any susceptible counterpart, therefore merely describing a model of bacterial spread within a population. The exclusion of papers is described in Fig. 1.

### Assessing the included papers

The vast majority of the models were population models (77%), while a small number were individual- or agent-based models. Only one was a nested model, in which individuals (pigs) and the bacterial populations inside them were modelled (Table 2).

**Table 2:** Model description – results of the information extracted from the selected studies

Modelled process	Model type	Population	Environment	Reference no.
Development of AMR	Individual based	Bacteria	Not specified	[66]
	Population	Bacteria	Human	[53, 67]
			In vitro	[19, 58]
			Pig	[14, 51]
			River	[17]
			Not specified	[16, 49, 68, 29, 18]
		Human	Not specified	[32]
	Other types^a^	Bacteria	In vitro	[59]
			Not specified	[69]
Spread of AMR	Agent based	Bacteria	Not specified	[57]
	Individual based	Dog	Hospital	[13]
	Population	Bacteria	Slurry	[64]
			Not specified	[30]
		Human	Community	[31, 48]
			Hospital	[47]
		Pig	Farm	[54]
Development and spread of AMR	Agent based	Bacteria	In vitro	[60]
	Nested	Pig & bacteria	Farm	[27]
	Population	Bacteria	River	[63]
		Human	Hospital	[33, 46, 50, 56]
			Hospital & community	[20]
			Not specified	[15, 34, 55]
	Other types^a^	Bacteria	In vitro	[61]
			Cellular automata	[70]

^a^Beverton-Holt [69] and Chemostat [59], Cellular automata [70], Chemostat [61]; *AMR* antimicrobial resistance

Analytical solutions were used in eight models, while numeric simulations were used in 16 models. In 14 models, analytical solutions were obtained using numeric simulations. There were 29 models for which the uncertainty was modelled deterministically, a further four were stochastic, and five used both stochastic and deterministic uncertainty display (Table 3).

**Table 3:** Modelling technique – results of the information extracted from the selected studies

Simulation or analytic	Uncertainty display	Reference no.
Analytic	Deterministic	[18, 29, 30, 34, 48, 49, 59, 61]
Simulation	Deterministic	[17, 19, 31, 33, 50, 54, 63, 64, 67, 70]
	Stochastic	[13, 51, 60, 66]
	Deterministic & stochastic	[20, 27]
Analytic and simulation	Deterministic	[14–16, 32, 46, 47, 53, 55, 58, 68, 69]
	Deterministic & stochastic	[57, 58]

It was not relevant to describe the mixing of the population for four of the papers, as they focused on development of AMR without population mixing. The majority of the models used a homogenous mixing of the population (25 papers, 66%). There was no conversion from resistant strain to sensitive or vice versa in seven of the published models (18%), of which five described only a single strain infection and five modelled more than one strain without conversion between resistant and sensitive strains (Table 4).

**Table 4:** Modelling pathway – results of the information extracted from the selected studies

Mixing of population	Co-existing level	Reference no.
Homogeneous	No conversion	[14, 51, 58]
	Single strain	[19, 29, 30]
	Uni-directional	[15, 31, 33, 46–48, 53, 57, 59, 64]
	Bi-directional	[18, 34, 50, 54–56, 66, 67]
Heterogeneous	No conversion	[16]
	Single strain	[60]
	Uni-directional	[70]
	Bi-directional	[13]
Homogeneous & heterogeneous	No conversion	[27]
	Uni-directional	[61]
	Bi-directional	[17, 20, 63]
Not relevant	Single strain	[69]
	Uni-directional	[32, 49, 68]

Phenotypic AMR in single strains was modelled in the majority (11 models, 29%) of the models. Only nine models included the effect of the immune system, and 11 of the models used a constant effect of antimicrobial compound (Table 5).

**Table 5:** Model specification – results of the information extracted from the selected studies

AMR display	Number of resistant strains	Bacterial species	Modelling of antimicrobial dosing	Antimicrobial compound	Immune system	Reference
Genotypic	Single	Not specified	Not constant	Single, not specified	Not relevant	[18]
Phenotypic	Single	Specified	Constant	Single, specified	Not relevant	[16]
				Multiple, specified	Yes	[32, 53]
			Not constant	Single, specified	No	[14, 34]
					Not relevant	[19, 58]
				Single, not specified	No	[13]
				Multiple, not specified	Yes	[15]
					No	[48]
					Not relevant	[64]
		Not specified	Constant	Single, specified	Not relevant	[30]
				Single, not specified	Yes	[49]
					No	[67]
			Not constant	Single, specified	Not relevant	[63]
				Single, not specified	Yes	[46, 70]
					No	[20, 31, 54]
					Not relevant	[57, 69]
				Multiple, specified	Not relevant	[17]
				Multiple, not specified	No	[35]
	Multiple	Specified	Constant	Single, not specified	No	[27]
			Not constant	Single, specified	No	[51]
				Multiple, specified	No	[47]
					Not relevant	[60]
		Not specified	Constant	Single, not specified	Not relevant	[61]
			Not constant	Multiple, not specified	Yes	[33, 50, 56]
					No	[55]
Geno- and phenotypic	Multiple	Not specified	Not constant	Single, not specified	Not relevant	[66]
Other	Single	Specified	Constant	Multiple, specified	Not relevant	[29]
		Not specified	Constant	Single, not specified	Not relevant	[59]
	Multiple	Not specified	Constant	Single, not specified	Not relevant	[68]

*AMR* antimicrobial resistance

Validation of the models was not reported in 27 of the papers (71%). Three models were validated based on the literature and ten models were validated based on data. Sensitivity analysis was carried out in 27 papers, while 11 papers did not report conducting a sensitivity analysis. Four of the papers reported no validation, sensitivity analysis or bifurcation analysis (Table 6).

**Table 6:** Model validation – results of the information extracted from the selected studies

Validation model	Sensitivity analysis	Bifurcation analysis	Reference
Data	Yes	Yes	[20]
		No	[13, 14, 16, 19]
	No	No	[29, 30, 58, 63]
		Not relevant	[60]
Literature	Yes	Yes	[15]
		No	[17, 18]
None	Yes	Yes	[46–48, 53, 64, 66–69]
		No	[27, 31–33, 49, 50, 54, 55–57]
	No	Yes	[55, 61]
		No	[34, 51, 59, 70]

### Model usefulness and documentation

The papers frequently lacked proper discussion and evaluation of the model assumptions, the usefulness of the data for input parameters and the implications of model conclusions in relation to real-life situations. Only eight papers [13–20] verified the model and conducted sensitivity analysis, thus complying with two of the TRACE criteria [9] (Criteria 6 and 7, Table 6). We identified three papers [14, 17, 18] that satisfied all TRACE criteria except element 5, as none of the studies confirmed the internal validity of the models. Furthermore, these three studies could have provided a better evaluation of the implications of data for input parameters and model assumptions (Table 7).

**Table 7:** Fulfilment of the TRACE elements

Study	Problem formulation	Model description	Data evaluation	Conceptual model evaluation	Implementation verification	Model output corroboration
Suthar et al., 2014 [13]	Yes	Yes	Yes	No	No	Yes
Nguyen et al., 2014 [14]	Yes	Yes	Yes	Yes	No	Yes
Ibargüen-Mondragón et al., 2016 [15]	Yes	Yes	No	No	No	No
Bhagunde, Nikolaou, and Tam, 2015 [16]	Yes	Yes	No	Not completely	No	Not completely
Hellweger, 2013 [17]	Yes	Yes	Yes	Yes	No	Yes
zur Wiesch, Engelstädter, and Sebastian Bonhoeffer, 2010 [18]	Yes	Yes	Yes	Yes	No	Yes
Tam et al., 2007 [19]	Yes	Yes	No	Not completely	No	Not completely
Kouyos, zur Wiesch, and Bonhoeffer, 2011 [20]	Yes	Not completely	Not completely	Not	No	Yes

For a complete description of the TRACE elements see Grimm et al. [9]. The two TRACE elements model output verification and model analysis were fulfilled by all eight studies as this was a selection criterion for the comparison with the TRACE elements

### Description of comparison papers

A special class of papers relating to quantitative comparisons of mathematical models of AMR was identified. Two papers compared the predictions of individual-based models [21] and stochastic models [22] to deterministic differential equation models. Both papers concluded that the deterministic approximation is valid when the number of simulated individuals is sufficiently large and the research question is not driven by single events (i.e. extinction events).

One paper [23] compared SIR models of four, six, eight and 12 compartments to include dual infection and time lag between treatment and AMR development. The inclusion of dual infections covers situations where patients may recover to a state with a coexistence of strains or strain takeover by the sensitive or resistant strain, depending on parameters. These results were independent of the complexity of the model.

One paper [24] compared six different deterministic differential pharmacodynamic models and the ability of statistical methods to identify data simulated from the six models as belonging to the correct one. They concluded that datasets containing only counts of bacteria did not provide sufficient information to identify the correct model. Additional experiments must be undertaken to determine which class of pharmacodynamic models best describe the data.

## Discussion

Recently, Heesterbeek et al. [25] reviewed the importance of mathematical modelling of infectious disease dynamics in terms of improving public health. The authors concluded that, mathematical models can provide inside that can be used in public health policies through the use of new data.

AMR is a major threat to public health, and the fight against it could benefit from the use of mathematical modelling. It could play an important role in providing an insight into the dynamics of AMR, quantifying the effect of factors that influence it and providing tools for its control and prevention. Furthermore, modelling can present an opportunity to elucidate potential gaps in our knowledge.

The reviewed papers varied in their choice of model structure and complexity – from simple deterministic models to advanced mechanistic models (agent-based, individual and nested models). However, they generally provided little justification for the model type and structure that was chosen. In addition, the majority of studies focused on modelling only one unit (Table 2), a single strain of a pathogen (Table 5), assumed homogeneous mixing (Table 4), and ignored uncertainty and stochasticity in the development and/or spread of AMR (Table 3). AMR is a multifactorial problem with several elements – including external factors and interactions within and between populations (microbiota, animal and human populations) – able to affect its development and spread [26]. This creates nonlinearity, heterogeneity, and stochasticity that should be considered when mathematical models of AMR are developed. Opatowski et al. [7] wrote that models should take into account the specific pathogen characteristics such as the resistance mechanism of the pathogen and cooperation among species. They concluded that this would provide major improvements of models.. However, in the 6 years since their review was published, only one paper has described a truly nested model [27] (Table 2), modelling multiple bacterial strains within individuals (pigs) that interact as a population with a heterogeneous structure. Unfortunately, this model was not validated and does not allow conversion of the pathogens. Furthermore, one article [28] published a framework to cope with multiple nested layers from the genetic composition of cells, to the environment of cells, the host of the cells, and the environment of the host. This type of models is clearly something to be striving for in the future as the AMR problem is highly complex, and the interaction on many levels require a deep understanding. It would also be very helpful if the community could commit to using this type of standard models, so that the huge work of parameterise these models could begin. That in the future we could stand on the shoulders of each other instead of trying to building new models for every single problem.

Mechanistic modelling using stochastic processes can describe complex heterogeneous structures and processes, multiple pathogens/genes simultaneously, and model biological interactions that may affect AMR such as the immune system, the dosing effect of antibiotics, the microbiome and variabilities involved in the system. In addition, these models can provide insights into the temporal dynamics of AMR, both in the individual and the population. Arepeva et al. [11] also point to the advantage of this class of models over simpler types of models such as deterministic differential equations. Nine models used analytical solutions to solve the modelled system (Table 2), providing extensive mathematical solutions with a limited interpretation of the applicability of the outcomes to real life. In fact, only two papers [29, 30] attempted to validate the models using data. Analytical solutions can be useful when trying to avoid time-consuming and computer-intensive simulations. Nevertheless, from a practical point of view, the high complexity of AMR and limited translation of analytical solutions to real life can call the usefulness of this approach to solve and/or limit the AMR problem into question.

Ideally, models of AMR should be validated by data. However, many of the published models represent hypothetical situations in hospitals or communities with no supporting data [31–35]. Such models are only useful in the event that a similar hospital or environment can be located. If this is the case, experiments or observational studies can be carried out to validate the models. In addition, there seems to be a lack of knowledge of how to implement different typical parameters and how to relate these to reality. For instance, what is the carrying capacity of a human patient for different types of AMR bacteria or genes, and how do levels of AMR relate to transmission rates under different circumstances in a hospital or community? This highlights the necessity for further fundamental and conceptual research to provide information and data to develop useful simulation models of AMR processes.

Validation is an essential factor when developing a mathematical model. Validation can be both internal (conducted to ensure that the model is doing what it should) and external (conducted to assess whether the model outcomes resemble real life). Models were externally validated in only 13 of the studies; ten studies used data and three were dependent on literature. The absence of validation in many of the published models (Table 5) could be due to a lack of usable data. There is a large gap in our knowledge when it comes to the dynamics of AMR inside a host, especially in terms of genotypic AMR. Interestingly, none of the studies indicated that internal validation had been conducted. Several methods can be used to internally validate the models, such as the rationalism method, tracing method and face validity [36]. Internal validation is important to ensure that the code is free from errors, satisfying the fifth criteria of the TRACE method [9]. It is possible that internal validation has been conducted, even if it is not mentioned in the paper. Nevertheless, we believe that it is important to describe the methods and steps used for internal validation in order to ensure confidence in the predictions. A lack of model validation may increase the risk of erroneous outcomes and conclusions, which in turn may reduce any confidence the scientific community and decision makers have in the predictions. Strict internal validation of the models must therefore be conducted and reported. Furthermore, additional research should be conducted to provide data to externally validate the models, resulting in models that can provide trustworthy recommendations. There exist papers on mathematical models where the TRACE criteria are fulfilled. A good example hereof is written by Foddai et al. [37].

The vast majority of the papers focused on modelling AMR in relation to humans, either directly by modelling human populations (in hospitals or communities) or in bacteria related directly to human health. Only four models relating to animals were conducted (Table 1). Animals might constitute a reservoir of AMR that can be spread to humans through their products (e.g. meat [38–41]), the environment (faeces used as fertilisers [41–43]), or direct contact [41, 44, 45], so more attention should be paid to improving our understanding of AMR dynamics within livestock production systems and the environment.

All studies included in this review report that an increase in antimicrobial use increases AMR in general. Some papers report that certain strategies show relatively smaller increases in AMR, which could be due to reducing contact rates or cycling different kinds of antimicrobial products [13, 15, 20, 27, 31–34, 46–56]. One paper reported a decrease in AMR when using an antimicrobial against which bacteria have no resistance [35]. However, as the authors report, such a property is transient and will diminish in time proportional to the extent to which that drug is used. Some papers construct several pathways to achieving AMR (i.e. hospital- versus community-acquired AMR) and deduce the parameter values at which the R0 (basic reproduction number, denoting how infectious the disease is) is above 1 [20, 31, 48, 52, 55, 35, 57]. However, no papers actually fit epidemiological data to determine parameters or validate their model. There are many studies looking at the epidemiological spread of specific resistant pathogens (e.g. MRSA), but these studies were excluded from this review, as we are interested in the spread of resistance rather than specific pathogens. In stating that there are no data of epidemiological spread, we mean spread of resistance between bacteria in an in vivo situation. There is an abundance of papers describing spread in in vitro experiments [19, 58–61], yet we believe that such parameters can at best be a starting point for estimating parameters in vivo, as the natural environment is much more complex and competitive than a petri dish.

To improve our understanding of AMR, we might need to specifically understand the mechanisms that generate resistance. Some papers in our review modelled specific mechanisms (i.e. efflux pumps, senescence, indoles, or influence of the normal flora) [29, 53, 59, 61]. However, only one of these papers was actually validated by data [29]. Modelling specific mechanisms might be a way to better understand the behaviours and interactions of bacteria using these methods, and it may also give us a better understanding of how AMR interacts when multidrug resistance is considered.

The environmental impact of AMR was modelled in four papers: three of the papers modelled rivers and described the accumulation and survival of AMR [17, 62, 63]. One of the papers also included the effects of metals on the development of AMR [17]. One paper described AMR bacteria growth in slurry [64], showing that AMR bacteria can thrive in this medium. The aggregation and possible growth of AMR bacteria in the environment might be of great concern if bacteria are exposed to a mixture of AM from several sources e.g. in rivers or slurry, they may acquire multiple resistances. If there is a chance that these can then transfer back into the animal or human population, these types of models may be very useful.

The studies originated from 16 different countries and were published in 30 different journals. This indicates that a relatively large number of journals are interested in modelling AMR. Furthermore, it highlights that mathematical modelling is a relevant subject for a broad section of the scientific community. When screening and excluding papers, we might have excluded papers based on a misinterpretation of the title or abstract. However, to minimise such mistakes, we strived to include papers in cases where there was any doubt.

The majority of the studies modelled phenotypic AMR, while few models represented genotypic AMR (Table 5). Modelling genotypic AMR can be more complicated as many genes can be linked to a specific antibiotic, and the relationship between gene abundance and antibiotic use seems to be more complex than first anticipated [65]. Modelling genotypic AMR requires the relevant genes for the modelled AMR to be represented, as well as circumstances allowing for the genetic AMR to be expressed as phenotypic AMR, leading to a spread of the resistant pathogen within the population. Published models of genotypic AMR do not link this AMR type to the development of phenotypic AMR and the subsequent spread of the resistant pathogen between individuals [18]. This is perhaps due to a lack of information on the necessary circumstances for the phenotypic expression of genetic resistance determinants, thus emphasising the need for more research to better understand this process. Understanding the process is essential in the prevention of AMR development and spread.

In our opinion, the following elements should be considered when developing future models of AMR:

1. Modelling the biological processes mechanistically. This allows heterogeneous processes and structures to be modelled and provides an insight into the ‘how and why’ of AMR occurrence, transfer and persistence.
2. Incorporating the uncertainty and variability of the system using stochastic modelling.
3. Extensive sensitivity analysis and model validation (both internal and external) using data that can support model development, parameterisation and validation.

The current study provides a comprehensive review of published models of AMR spread and development since 2006. Although the study focuses on providing insights into the technical elements of and differences between the models, it also provides an insight into the elements that should be included when AMR is modelled.

## Conclusions

Many mathematical models of AMR development and spread exist. However, there is still a lack of knowledge regarding the underlying mechanisms at work, thus limiting the true usefulness of the developed models. Furthermore, few models complied with the TRACE criteria. Future AMR models should elucidate the dynamics and variability of AMR occurrence and spread in order to investigate ways of effectively influencing these dynamics to prevent and control AMR. In addition, it is of utmost importance to focus research on providing data to parameterise and validate AMR models in order to extract useful conclusions from them. There is a need for more rigorous model development and testing and more abundant experimental and observational data to support model validation.

## Abbreviation

AMR: Antimicrobial resistance
